# Genetics Dissection of Complex Traits in the Genomic Era

**DOI:** 10.2174/138920212800543075

**Published:** 2012-05

**Authors:** Bernardo Ordas

There is a renewed interest for Quantitative genetics since the genomic revolution. Quantitative or complex traits are those controlled by multiple genes so that the inheritance of such complex traits is non-Mendelian, although each individual gene shows Mendelian inheritance. With the advent of genomic tools, for example Next Generation Sequencing (NGS) and deep NGS sequencing of RNA transcript (reviewed in the chapter of Ana Perez de Castro *et al*. with a specially focus on plants) scientists have access to information that was unimaginable some decades ago. However, it is essential to recognize that the objectives of geneticists remain the same, although the technology allows the answer to questions that some years ago were unthinkable. With the myriad of statistical methods, experimental designs and phenotypic and molecular data that are now available a lucid and comprehensive vision of the objectives, findings and challenges facing nowadays by geneticists is more needed than ever. 

As explained by William Hill, one of the main practical objectives of quantitative genetics is an accurate prediction of breeding values because the rate of genetic improvement is proportional to the accuracy of those estimations. The author showed that animal breeders have been ahead in the development of prediction models with the “animal model” for phenotypic data and they are again the first in the race with the principles and methods for “Genomic selection” for genomic data. The basic idea of genomic selection is to fit all the markers and assume they are associated through linkage disequilibrium (LD) with genes with an effect on the trait. Jack Dekkers, one of the pioneers of the application of genomic selection, indicates in his chapter that genomic selection increases the rate of genetic improvement not only by increasing the accuracy of breeding values, but also by reducing the generation interval. The author classifies the statistical methodologies into two main groups: Bayesian with a more or less arbitrary choice of prior distribution of gene effects and BLUP mixed models (GBLUP) which typically assume that genetic variance is distributed equally across all SNPs. For the SNP density used nowadays experimental results show similar prediction ability for Bayesian and for GBLUP; however, the SNP density is increasing as genotyping costs are dropping, and at higher SNP densities the Bayesian methods are expected to have higher prediction ability than GBLUP methods. Genomic selection only recently received attention from plant breeders, although, as discussed by Perez de Castro *et al*., an article with preliminary data from Arabidopsis, maize and barley show higher efficacy of the method compared to conventional marker assisted selection. 

The study of complex traits in humans has a different final objective than in animals or plants: prediction of genetic risks to diseases rather than genetic improvement of the populations. However, the genetic approach is very similar and the main relevant question remains to predict the genotypic value of individuals. For the development of risk predictors for complex diseases the area under the receiver operator characteristics (ROC) curve can be used to assess the discriminative ability of a prediction model. Suzanne Rowe and Albert Tenesa, as explained in their chapter about human genetics, simulated data for different diseases and found that the discriminative ability is higher when all markers all included in the analysis, although the accuracy of prediction is very dependent on the underlying genetic architecture of the trait. This is in agreement with the principles of genomic selection, although the success of prediction methods for complex diseases will be limited by the disease prevalence and the heritability. 

Regarding the elucidation of the genetic architecture of quantitative traits, according to Hill, one of the more precise designs to analysis quantitative trait loci (QTL) is “association mapping” because it uses populations with low LD which increases the resolution for detecting QTLs. If animal breeders were the first to successfully apply genomic selection, human geneticists have carried out the more complete association mapping experiments until now. Rowe and Tenesa indicate that, by means of genome wide association studies (GWAS), there are more than 90 cancer loci susceptibility loci identified, 39 for type 2 diabetes and 71 for Crohn disease. According to the authors, most large common variants have now been discovered, although a huge amount of the genetic variability remains hidden; therefore the crucial question is how to capture the remaining variation. Dekkers performs an interesting analysis in layer chickens in which the genome was divided into 1000 windows of 1 Mb and the genetic variance of each window was estimated. The main conclusion is that over 50 % of the genetic variance resides in a large number of genomic regions spread across the genome approaching the infinitesimal model of quantitative genetics. The complexity of a huge number of genes together with pleotropic effects, which also could play a significant role according to some experiments discussed by Hill and by Rowe and Tenesa, make the study of the quantitative traits a very challenging task. For that reason, as pointed out for most of the authors of the issue, the underlying biological mechanisms remain elusive for most traits.

If things look quite complicated with only genetic effects averaged over environments, genotype × environment (GE) interaction adds further complexity to our understanding of genetic variation. The main GE models with their advantages and disadvantages are reviewed and discussed in great detail by Jose Crossa who himself has developed several GE models. The author presents, for example, the factorial regression (FR) model which allows incorporating external environmental and genotypic variables into the model and is useful for finding the climatic causes of GE or for estimating effects and locations of Quantitative traits loci (QTL) and QTL × environment interaction (QEI). A multi-trait multi-environment (MTME) mixed model is also presented by Crossa with an example that illustrates the importance of studying jointly, not only different environments, but also different traits. The author indicates that most genomic selection methods are environment-specific and points out the need for the development of new multi-environment and multi-trait models for genomic selection. The author himself described the use of biplots and a LASSO Bayesian model to studied GE at the level of estimated marker effects.

It is clear from the chapters of this issue that Quantitative genetics continue to be an active area of research with many groups working on the development of better human risk predictors or breeding values estimators using genomic information. Nowadays, one of the main challenges of quantitative geneticists is the integration and interpretation of the huge amount of information that is generated by new technologies. There is a great expectation for the possibilities of systems genetic for elucidating the genetic architecture of Quantitative traits, although time will tell if systems genetic meet the expectations. Anyway, as pointed out by one the author of this issue, the true is that much of the work it is still to be realised.

I acknowledge Bill Hill for his invaluable help in the preparation of this issue. I would like also to acknowledge Bruce Walsh, Daniel Gianola and other anonymous referees that have improved its quality.

